# Artificial Vagina

**Published:** 1915-12

**Authors:** 


**Artificial Vagina.** W. Douglas Ward, Rochester, N. Y., Surg., Gyn. & Obs., Nov., considers operation imperative if the uterus is functionating, and important for psychic reasons in the majority of all cases, even if no menstrual molimen occurs. Earlier plastic operations with skin flaps or peritoneum from Douglas's cul-de-sac, were mostly failures. Gersuny, 1897, took flaps from the rectum with success so far as the maintenance of a canal was concerned, but it was necessary to use a pessary. Baldwin of Columbus devised the present satisfactory method of using a portion of the ileum in 1904, but did not have opportunity to put it into execution till 1907. In 1912, he reported 6 successful cases. 7 other successful cases have been reported, including that of Thew Wright of Buffalo, (Buffalo Medical Journal, Dec. 1913). The author adds another successful case, a girl of 13, tarry blood to the amount of an ounce being found in the uterus. The Germans prefer to work with the lower bowel. Albrecht has constructed three vaginae from sigmoid, Schubert and Strassmann 9 cases from the rectum.

